# Identification of novel therapeutic target and prognostic biomarker in matrix metalloproteinase gene family in pancreatic cancer

**DOI:** 10.1038/s41598-023-44506-8

**Published:** 2023-10-11

**Authors:** Hong Luan, Linge Jian, Yuyan Huang, Yutong Guo, Liping Zhou

**Affiliations:** 1 Department of Laboratory Medicine, The First Hospital of China Medical University, Shenyang, 110001 Liaoning People’s Republic of China; 2 West China Medical School, Sichuan University, Chengdu, 610041 Sichuan People’s Republic of China; 3 Department of Clinical Laboratory Diagnostics, China Medical University, Shenyang, 110001 Liaoning People’s Republic of China; 4 GCP Center, The First Hospital of China Medical University, No. 155, Nanjingbei Street, Heping District, Shenyang, 110001 Liaoning People’s Republic of China

**Keywords:** Cancer microenvironment, Cancer therapy, Gastrointestinal cancer, Tumour biomarkers, Tumour immunology

## Abstract

Matrix metalloproteinases (MMPs) play an essential role in various physiological events. Recent studies have revealed its carcinogenic effect in malignancies. However, the different expression patterns, prognostic value, and immunological value of MMPs in pancreatic ductal adenocarcinoma (PDAC) are yet to be comprehensively explored. We utilized Gene Expression Profiling Interactive Analysis (GEPIA) and Gene Expression Omnibus databases to explore the abnormal expression of *MMPs* in PDAC. Then, Kaplan–Meier survival curve and Cox regression analysis were performed to assess the prognostic value of *MMPs*. Association between *MMPs* expression and clinicopathological features was analyzed through UALCAN website. Functional annotations and GSEA analysis were performed to excavate the possible signaling pathways involving prognostic-related *MMP*. TIMER and TISCH database were used to performed immune infiltration analysis. The expression of prognostic-related *MMP* in pancreatic cancer cell lines and normal pancreatic cells was detected by Real time quantitative PCR. We observed that 10 *MMP* genes were consistently up-regulated in GEPIA and GSE62452 dataset. Among them, five highly expressed *MMPs* (*MMP1, MMP3, MMP11, MMP14, MMP28*) were closely related to poor clinical outcomes of PDAC patients. Cox regression analysis indicated *MMP28* was a risk factor influencing the overall survival of patients. In the clinicopathological analysis, up-regulated *MMP28* was significantly associated with higher tumor grade and the mutation status of TP53. GSEA analysis demonstrated that high expression of *MMP28* was involved in “interferon_alpha_response” and “P53_pathway”. Immune infiltration analysis showed that there was no correlation between *MMP28* expression and immune cell infiltration. Single-cell sequencing analysis showed *MMP28* has strong correlations with malignant cells and stromal cells infiltration in the tumor microenvironment. And *MMP28* was highly expressed in various pancreatic cancer cell lines. In conclusion, *MMP28* may represent a potential prognosis biomarker and novel therapeutic molecular targets for PDAC.

## Introduction

Pancreatic ductal adenocarcinoma (PDAC), deriving from either ductal or acinar cells of the exocrine portion, constitutes more than 90% of all pancreatic cancers^1^. Despite its low incidence rate, the prognosis of PDAC is extremely poor, with a 5-year relative survival rate of only 9%^2^. According to the GLOBOCAN 2020 report, pancreatic cancer accounted for almost as many new cases (495,773) as cancer deaths (466,003) worldwide in 2020^3^. The latest study from the European Union countries and the UK showed that pancreatic cancer has moved up to the third leading cause of death among carcinomas, following lung and colorectal cancer^4^. Surgical resection combined with systemic chemotherapy remains the mainstay of treatment and the only possible curative approach^5,6^. Unfortunately, most patients with PDAC have already reached an advanced stage when diagnosed, with a low surgical resection probability. Although immunotherapy has shown effectiveness in treating several solid tumors^7,8^, the therapeutic benefits of immunotherapy such as anti-CTLA-4 and anti-PD-1 remain very limited in PDAC. Hence, exploring potential functions of known molecules and searching useful biomarkers could provide new therapeutic targets and prognostic biomarkers.

The matrix metalloproteinase (MMP) family, also known as matrixins, consists of 24 zinc-dependent endopeptidases in humans^9^. These proteases are capable of degrading various proteins in the extracellular matrix and regulating the release or activation of chemokines, cytokines, cytoskeletal proteins, and growth factors, thus affecting many fundamental physiological events, for instance embryogenesis, inflammation, angiogenesis, bone remodeling, and tumor growth and metastasis^10–13^. As early as the 1990s, the broad-spectrum MMP inhibitor (MMPI) batimastat was shown to inhibit breast cancer regrowth and metastasis in mouse xenograft model^14^. However, in clinical trials, broad-spectrum MMPI has not proved successful, mainly due to the bidirectional role of MMPs under pathological conditions, in which MMPs have both promoting and anti-tumor effects^15–17^. Recently, with the further understanding of biological activities of MMPs, narrow-spectrum MMPIs that are safer and more selective have been developed in cancer treatment^17,18^.

In the present study, we conducted a systematic and comprehensive analysis for all 24 human MMPs, aiming to identify suitable subtypes of MMPs as potential prognosis biomarkers and novel therapeutic targets in PDAC. We first screened MMP expressions, among which 10 genes were highly expressed in PDAC based on both Gene Expression Profiling Interactive Analysis (GEPIA) and Gene Expression Omnibus (GEO) databases. Then, by applying Kaplan–Meier survival curves and Cox regression analysis, we found that *MMP28* was a risk factor influencing the overall survival of PDAC patients. The relationship between MMP expression and the clinicopathological characteristics of PDAC patients was investigated using the UALCAN website. Moreover, Gene Ontology (GO) term enrichment, Kyoto Encyclopedia of Genes and Genomes (KEGG) pathway analysis, and Gene Set Enrichment Analysis (GSEA) were performed to excavate the possible signaling pathways involving *MMP28*. We used TIMER and the TISCH database to evaluate the link between *MMP28* and immune cell infiltration of tumors. Lastly, expression of *MMP28* in pancreatic cancer cell lines and normal pancreatic cells was determined using real*-*time quantitative PCR (RT-qPCR).

## Materials and methods

### Differential expression analysis of MMPs in PDAC

The GSE62452 dataset^19^ from the Gene Expression Omnibus (GEO) database (https://www.ncbi.nlm.nih.gov/geo/)^20^ was downloaded on December 5, 2022 to screen the expression of MMPs in PDAC tissues. Differences between the normal and tumor groups were statistically evaluated using the Mann–Whitney U test in GraphPad Prism 8.0.2 (GraphPad Prism Software Inc., San Diego, CA, USA), with *P* < 0.05 considered significant. The overall flowchart for the strategies and methods used in this study was shown in supplementary Fig. S1.

GEPIA (http://gepia.cancer-pku.cn/index.html) is a powerful web server for analyzing and visualizing the RNA sequencing expression data from The Cancer Genome Atlas (TCGA) and the Genotype-Tissue Expression (GTEx) projects^21^. In this study, we used GEPIA to identify the expression of MMP family genes in the TCGA and GTEx data. The screening criteria follow: |log (FC)|> 1 and *P* < 0.05.

### Survival analysis

Based on RNA-seq data, GEPIA was further used to verify the survival analysis. To explore the prognostic value of MMPs in PDAC patients, disease-free survival (DFS) and overall survival (OS) were acquired from GEPIA. For each gene, patients were classified into high- and low-expression groups, as defined by the median value, and survival curves were estimated using the Kaplan–Meier method. Hazard ratio (HR) with a 95% confidence interval (CI) was shown in the survival plot and *P* < 0.05 was considered significant.

### Construction and evaluation of the prognostic risk model

The mRNA expression profiles and corresponding clinical data for PDAC patients were retrieved from the TCGA data portal on December 5, 2022 (https://tcga-data.nci.nih.gov/tcga/). A total of 177 PDAC cases with complete survival information were selected. To ensure data quality in our analysis, five mucinous adenocarcinoma patients, eight neuroendocrine carcinoma patients, and six patients with less than 1-month survival time were excluded from the trial. Univariate Cox regression analysis was performed for screening the prognosis-related genes, with *P* < 0.05 considered significant. Multivariate Cox regression analysis was used to identify significant prognostic signature; those genes with *P* < 0.05 were used in follow-up analysis. Cox regression analysis was used for the identification of prognosis-related MMPs most correlated with overall survival using the “survival” package for R (version 4.0.3). The risk score was calculated according to the following formula: risk score = exp1*β1 + exp2*β2… + expi*βi (expi, gene expression level; βi, coefficients of the multivariate Cox analysis). All patients in the TCGA cohort were divided into low- and high-risk groups based on the cut-off values of median risk scores, and the survival differences between two groups were compared by the Kaplan–Meier log-rank test. The “survival”, “randomForestSRC” and “timeROC” R packages were employed to perform a 3-year receiver operating characteristic (ROC) analysis. The prognostic efficiency of the model was measured by the area under the ROC curve (AUC). Subsequently, combination with the clinical features, such as age, sex, tumor grade, and TNM stage, the independent prognostic value of the risk score was further analyzed by univariate and multivariate Cox regression analyses, with *P* < 0.05 considered significant.

## Clinicopathological parameters correlation with prognosis-related MMPs

The University of Alabama at Birmingham cancer data analysis portal (UALCAN) (http://ualcan.path.uab.edu/analysis.html) is an easy-to-use web-based tool for performing in-depth analysis of TCGA transcriptome and clinical patient data^22,23^. For this study, we analyzed the expression profiles of *MMP*s in PDAC samples based on tumor stage, grade, lymph node metastasis, and *TP53* mutation status using the UALCAN database. Box–whisker plots were used to illustrate the expression of prognosis-related MMPs in subgroups of pancreatic cancer samples. Differences between the two groups were determined using Student’s *t*-test, with *P* < 0.05 considered significant.

### Functional enrichment analysis

Genes co-expressed with *MMP28* were analyzed via the UALCAN website, and the top 20 genes positively correlated with *MMP28* in PDAC (Pearson correlation coefficient > 0.5) were selected to perform function enrichment analysis. Metascape (https://metascape.org/gp/index.html#/main/step1) is a popular portal, which combines functional enrichment, interactome analysis, gene annotation, and membership^24^. In this study, the Metascape database (v3.5) was applied for the pathway enrichment analysis to investigate the distribution of 20 similar genes within the KEGG and GO databases^25–27^. GeneMANIA (http://www.genemania.org), a user-friendly website, can display gene lists that share the same functions as submitted genes and provide a protein–protein interaction network^28^. We predicted the function and interaction of 20 similar genes using GeneMANIA.

### GSEA analysis

To further investigate the underlying biological activities of *MMP28*, GSEA analysis was implemented in the Broad Institute desktop application (version 4.1.0). Samples in the TCGA-PAAD dataset were classified into two groups (high and low expression) based on the average expression of *MMP28*. The Molecular Signatures Database (MSigDB) was used as follows: h.all.v7.5.symbols.gmt, c2.cp.kegg. v7. 5.symbols.gmt, and c5.all.v7.5.symbols.gmt, with all other parameters in GSEA software set at default. The normalized enrichment score (NES) > 1.5, nominal (NOM)*P*-value < 0.05, and false discovery rate (FDR) q-value < 0.25 were defined as the significantly enriched gene sets.

### Immune infiltration analysis

The TIMER web server (https://cistrome.shinyapps.io/timer/) is a visual resource for comprehensive analysis of immune infiltrates across diverse cancer types^29,30^. The correlation between prognosis-related *MMP* expressions and a large number of immune infiltrating cells (B cells, CD4 + T cells, CD8 + T cells, neutrophils, macrophages, and dendritic cells) in PDAC were analyzed in the “Gene” module using TIMER, and scatterplots were generated and displayed the purity-corrected partial Spearman’s rho value and statistical significance.

The Tumor Immune Single-Cell Hub 2 (TISCH2) (http://tisch.comp-genomics.org/) is a single-cell RNA-seq database providing detailed cell-type annotation at the single-cell level focusing on tumor microenvironment of different cancer types^31^. In this study, eight datasets (PAAD_CRA001160, PAAD_GSE111672, PAAD_GSE141017, PAAD_GSE148673, PAAD_GSE154778, PAAD_GSE158356, PAAD_GSE162708, and PAAD_GSE165399) were enrolled to analyze the correlation between *MMP28* expression and abundance of immune cell infiltration.

### Expression levels of *MMP28* in tumor cell lines

The *MMP28* expression levels in different tumor cell lines and pancreatic cancer cell lines were obtained from the Cancer Cell Line Encyclopedia (CCLE) database (https://portals.broadinstitute.org/ccle), and were visualized using the R package ggplot2.

Cell lines HPDE6-C7, BxPC3, SW1990, and PANC-1 were purchased from Shanghai Enzyme Research Biotechnology Co., Ltd. (Shanghai, China). All cell lines were maintained in Dulbecco’s Modified Eagle Medium (Gibco, USA) and supplemented with 10% fetal bovine serum (Sigma, USA), 100 U/mL penicillin, and 100 μg/mL streptomycin (Gibco, USA) at 37 °C with 5% CO_2_.

Total RNA of HPDE6-C7, BxPC3, PANC-1, and SW1990 cells was isolated using TRIzol (Invitrogen, USA) according to the manufacturer’s instructions. The complementary DNA (cDNA) were prepared using the PrimeScript RT Reagent Kit with gDNA Eraser (TaKaRa, Dalian, China). The RT-qPCR was performed using TB Green Premix Ex Taq™ (Tli RNaseH Plus) (TaKaRa) on an ABI QuantStudio 3 PCR system (Applied Biosystems, CA, USA). The gene for glyceraldehyde 3-phosphate dehydrogenase (GAPDH) was used as an internal reference. The primers used are listed in Supplementary Table S1. The amplification reaction included the following steps: 95 °C for 30 s, followed by 40 cycles of 95 °C for 10 s and 60 °C for 30 s. Relative mRNA expression was calculated using the relative quantification (2^−∆∆Ct^) method.

## Results

### Expression profile of matrix metalloproteinases in PDAC

We first utilized GEO dataset GSE62452 to obtain the differential expression of the *MMP* family in pancreatic cancer tissue and adjacent pancreatic non-tumor tissue. As shown in Fig. S2, of the 23 *MMP*s evaluated (*MMP18* was not detected in GSE62452), 12 genes were significantly up-regulated in pancreatic cancer tissues (*MMP1*, *MMP2*, *MMP3*, *MMP7*, *MMP8*, *MMP9*, *MMP10*, *MMP11*, *MMP12*, *MMP13*, *MMP14*, and *MMP28*), while seven genes were significantly down-regulated (*MMP17*, *MMP20*, *MMP21*, *MMP23B*, *MMP24*, *MMP25*, and *MMP26*). Other members of the *MMP* family (*MMP15*, *MMP16*, *MMP19*, and *MMP27*) showed no differential expression between pancreatic normal and cancer tissues.

**Figure 1:**
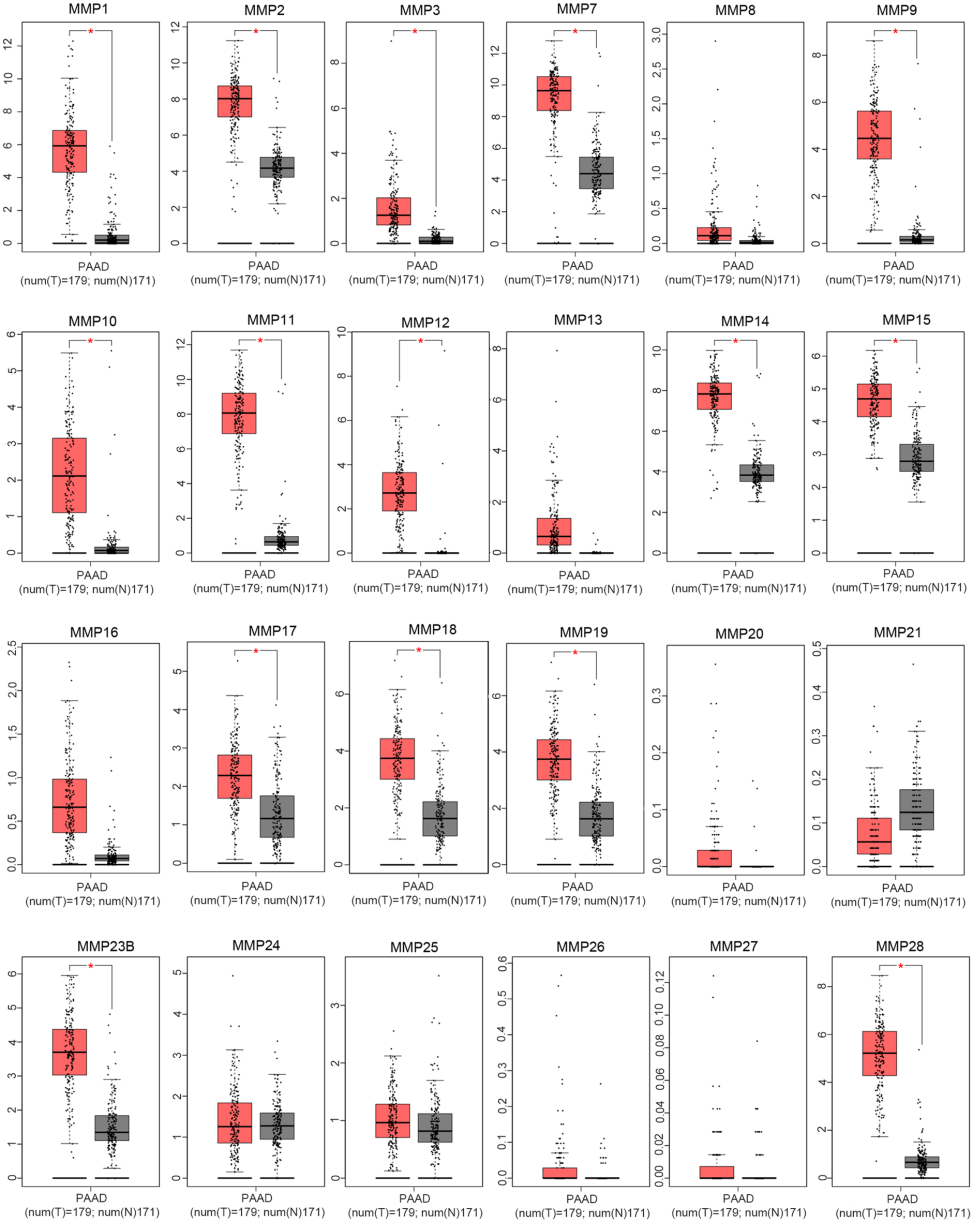
*MMP* expression profile in PDAC based on GEPIA database. The transcription levels in TCGA pancreatic tumors (n = 179) and matching normal tissue (n = 171) from the TCGA and GTEx databases. *P* < 0.05 is considered significant.

The online database GEPIA was used to verify the expression pattern of *MMP*s in PDAC (Fig. 1). The expression levels of 15 *MMP*s (*MMP1*, *MMP2*, *MMP3*,* MMP7*, *MMP9*, *MMP10*, *MMP11*, *MMP12*, *MMP14*, *MMP15*, *MMP17*, *MMP18*, *MMP19*, *MMP23B*, and *MMP28*) were obviously elevated in PDAC samples compared with matching TCGA and GTEx normal samples. In summary, 10 genes were consistently up-regulated in GEPIA and GSE62452, and were selected as potential targets for subsequent study: *MMP1*, *MMP2*, *MMP3*,* MMP7*, *MMP9*, *MMP10*, *MMP11*, *MMP12*, *MMP14*, and *MMP28*.

### Prognostic value of aberrant expression of MMPs in PDAC

**Figure 2:**
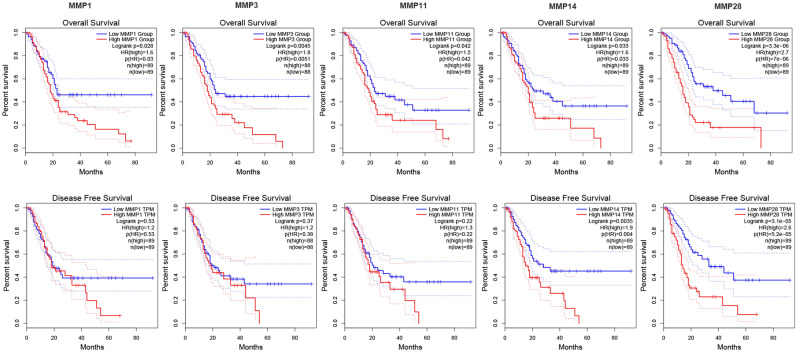
Prognostic value of *MMP* expression in PDAC patients (GEPIA). Overall survival and disease-free survival of five differentially expressed *MMP*s (*MMP1*, *MMP3*, *MMP11*, *MMP14*, and *MMP28*) in patients with PDAC.

To determine the impact of *MMP* expression on PDAC patient prognosis, we carried out OS and DFS analysis for selected *MMP*s based on TCGA data using the GEPIA database. In the OS analysis, overexpression of *MMP1*, *MMP3*, *MMP11*, *MMP14*, and *MMP28* was strongly correlated with poorer survival (Fig. 2). In the DFS analysis, high *MMP14* (*P* = 0.004) and *MMP28* (*P* = 5.2e−05) expression was remarkably related to poor DFS in PDAC patients. Expressions of *MMP2*, *MMP7*, *MMP9*, *MMP10*, and *MMP12* were not associated with OS and DFS of PDAC patients (supplementary Fig. S3). These results indicated that five differentially expressed *MMP*s (*MMP1*, *MMP3*, *MMP11*, *MMP14*, and *MMP28*) may serve as potential prognostic biomarkers for patients with PDAC.

### Construction of the prognostic model and identification of *MMP28* as an independent prognostic factor in PDAC

**Figure 3:**
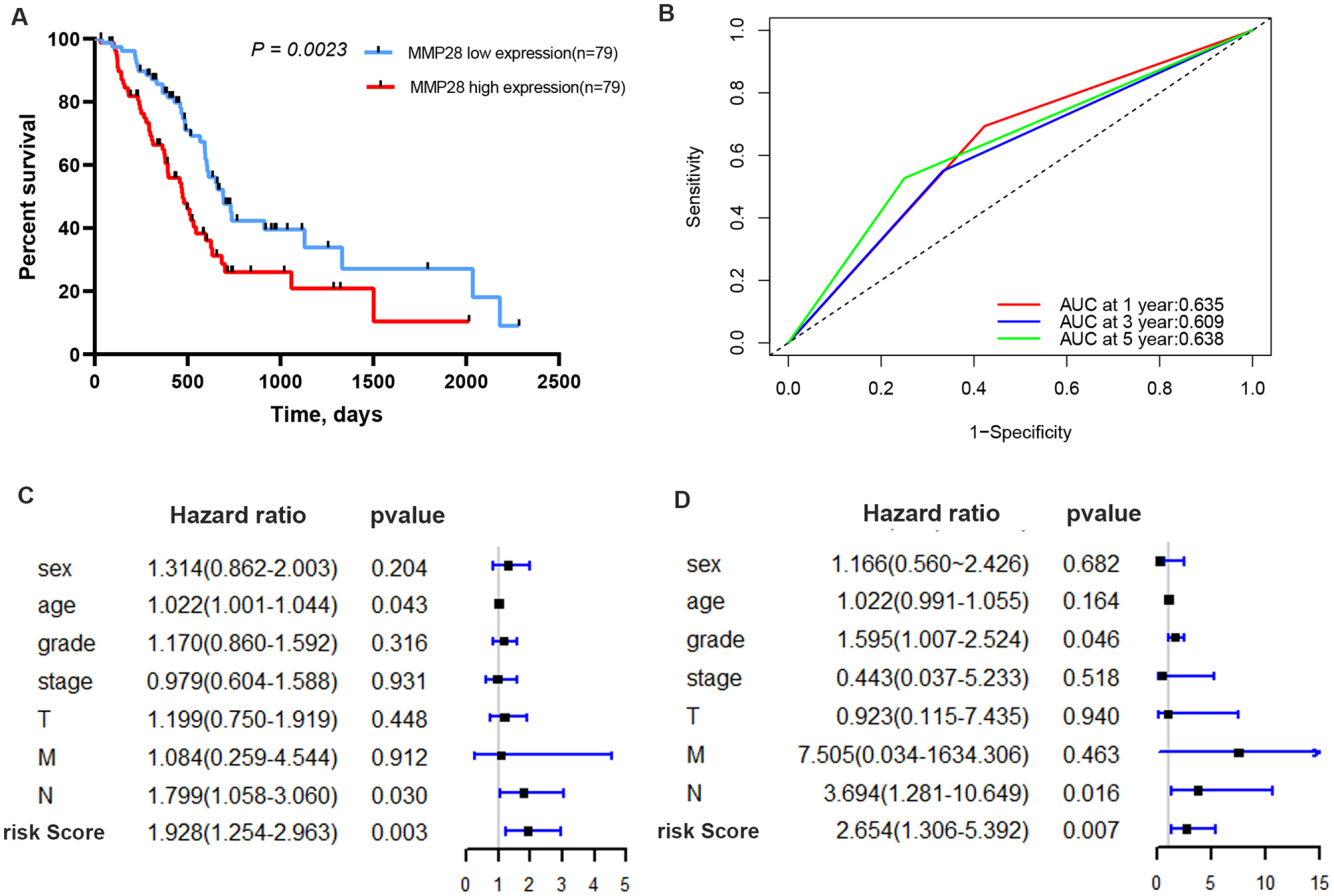
Validation of the risk model in the TCGA cohort. (**A**) Kaplan–Meier survival analysis for comparison of the OS between low- and high-risk groups based on the median risk score. (**B**) Time‐dependent ROC analysis of *MMP28*. (**C**) Univariate Cox regression analysis for the TCGA cohort. (**D**) Multivariate Cox regression analysis for the TCGA cohort. Factors include sex, age, grade, stage, and risk score. Hazard ratio > 1 represents a risk factor while < 1 represents a protective factor.

To further validate the prognostic value of *MMP*s, we carried out Cox proportional hazard regression on the expression profiles of 10 candidate *MMP*s; only *MMP28* was significantly (*P* < 0.05) correlated with patient survival (Table 1). The risk score was calculated as follows: risk score = 1.378e−2 × *MMP28* expression. The performance of the risk score was evaluated in the TCGA-PAAD dataset by dividing the patients into high- and low-risk groups using the median risk score as a cut-off threshold. The Kaplan–Meier curve indicated lower survival time for patients in the high-risk compared with the low‐risk group (*P* < 0.05) (Fig. 3A). The AUCs for 1-, 3-, and 5-year OS were 0.635, 0.609, and 0.638, respectively (Fig. 3B), indicating the favorable prediction performance of *MMP28* in PDAC patients.

**Table 1:** Univariate and multivariate analyses for overall survival of 10 differentially expressed *MMP*s in TCGA-PAAD cohort.

Gene	Univariate analyses			Multivariate analyses		
	Hazard ratio	95% CI	*P* value	Hazard ratio	95% CI	*P* value
MMP1	1	1–1	0.56	1	1–1	0.51
MMP2	1	1–1	0.59	1	1–1	0.97
MMP3	1.01	0.99–1.03	0.38	1.02	0.97–1.06	0.47
MMP7	1	1–1	0.87	1	1–1	0.79
MMP9	1	0.99–1.01	0.84	1	0.99–1.01	0.61
MMP10	0.99	0.95–1.04	0.69	0.99	0.94–1.04	0.7
MMP11	1	1–1	0.95	1	1–1	0.38
MMP12	1.01	0.99–1.03	0.55	0.99	0.95–1.04	0.8
MMP14	1	1–1	0.28	1	1–1	0.28
MMP28	1.01	1–1.02	0.01	1.01	1–1.02	0.01

To further identify whether *MMP28* could serve as an independent prognostic factor, univariate and multivariate Cox regression analyses were performed using TCGA-PAAD cohort with complete clinical information. Univariate analysis demonstrated that age (HR = 1.022, 95% CI = 1.001–1.044, *P* = 0.043), lymphatic invasion (HR = 1.799, 95% CI = 1.058–3.060, *P* = 0.030), and *MMP28* expression (HR = 1.928, 95% CI = 1.254–2.963, *P* = 0.003) were the risk factors influencing OS. The multivariate analysis showed that grade (HR = 1.595, 95% CI = 1.007–2.524, *P* = 0.046), lymphatic invasion (HR = 3.694, 95% CI = 1.281–10.649, *P* = 0.016), and *MMP28* expression (HR = 2.654, 95% CI = 1.306–5.392, *P* = 0.007) were independent risk factors for OS (Fig. 3C,D). Combined, these data suggest that *MMP28* could serve as a biomarker for prediction of OS among PDAC patients.

### Correlation of *MMP28* expression with clinicopathological parameters in PDAC

**Figure 4:**
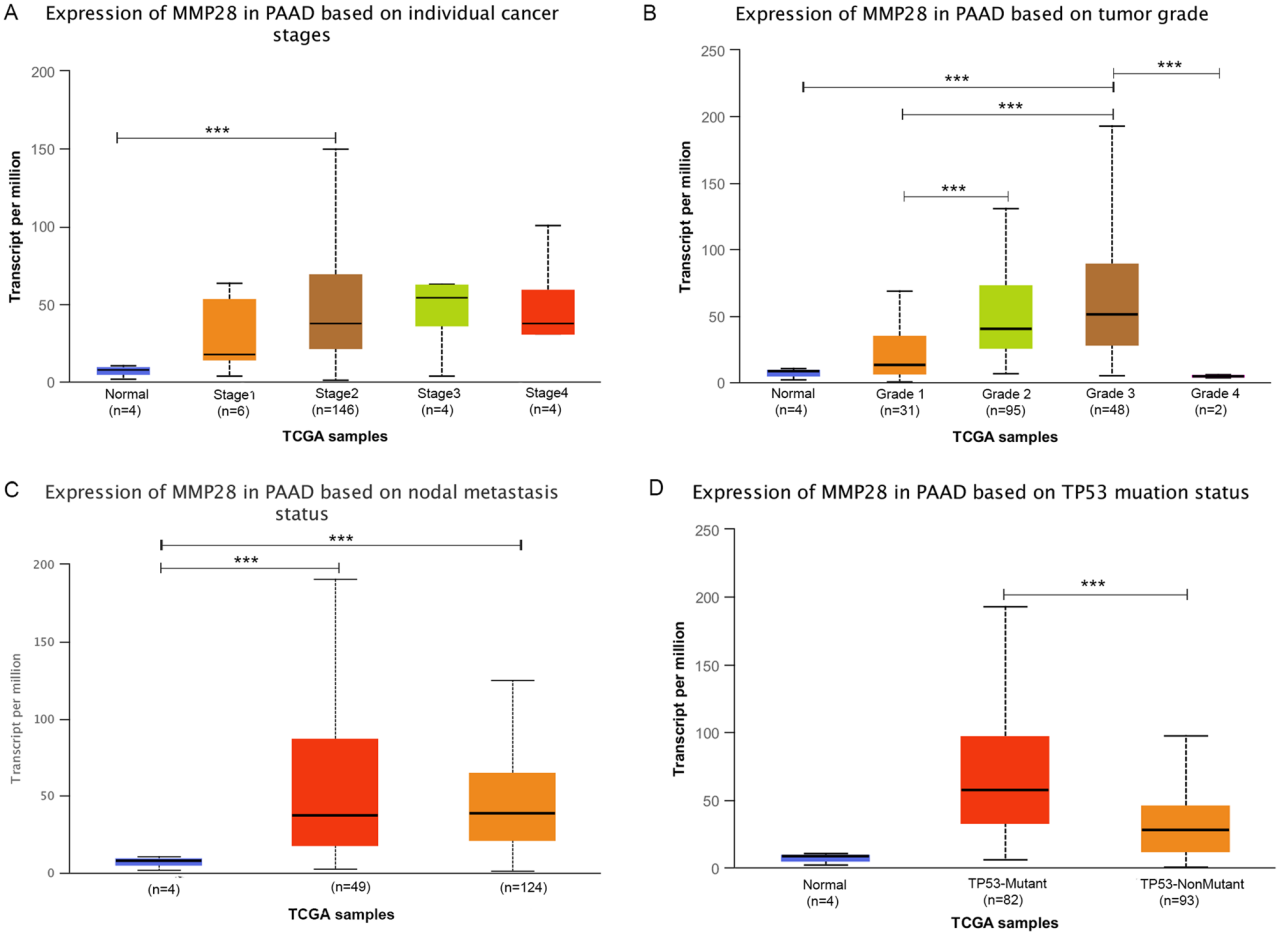
Correlation between *MMP28* expression and clinicopathological parameters in PDAC patients (UALCAN database). Tumoral *MMP28* level in PDAC patients with different (**A**) stages, (**B**) grades, (**C**) lymph node metastasis status, and (**D**) *TP53* mutation status. *P < 0.05; **P < 0.01; ***P < 0.001.

The relationship between *MMP28* expression and pancreatic cancer progression was investigated using the UALCAN database. Subgroup analysis demonstrated that the mRNA expression of *MMP28* was closely related to tumor grade and, as tumor grade increased, the *MMP28* expression level increased (Fig. 4B). However, there were no significant differences in *MMP28* expression level with cancer stage or nodal metastasis status (Fig. 4A,C). We then found that *TP53* mutation was significantly correlated with *MMP28* expression level. The PDAC samples had higher mRNA expression of *MMP28* with *TP53* mutation compared to those without (Fig. 4D).

### Functional analysis based on *MMP28* and similar genes in PDAC

**Figure 5:**
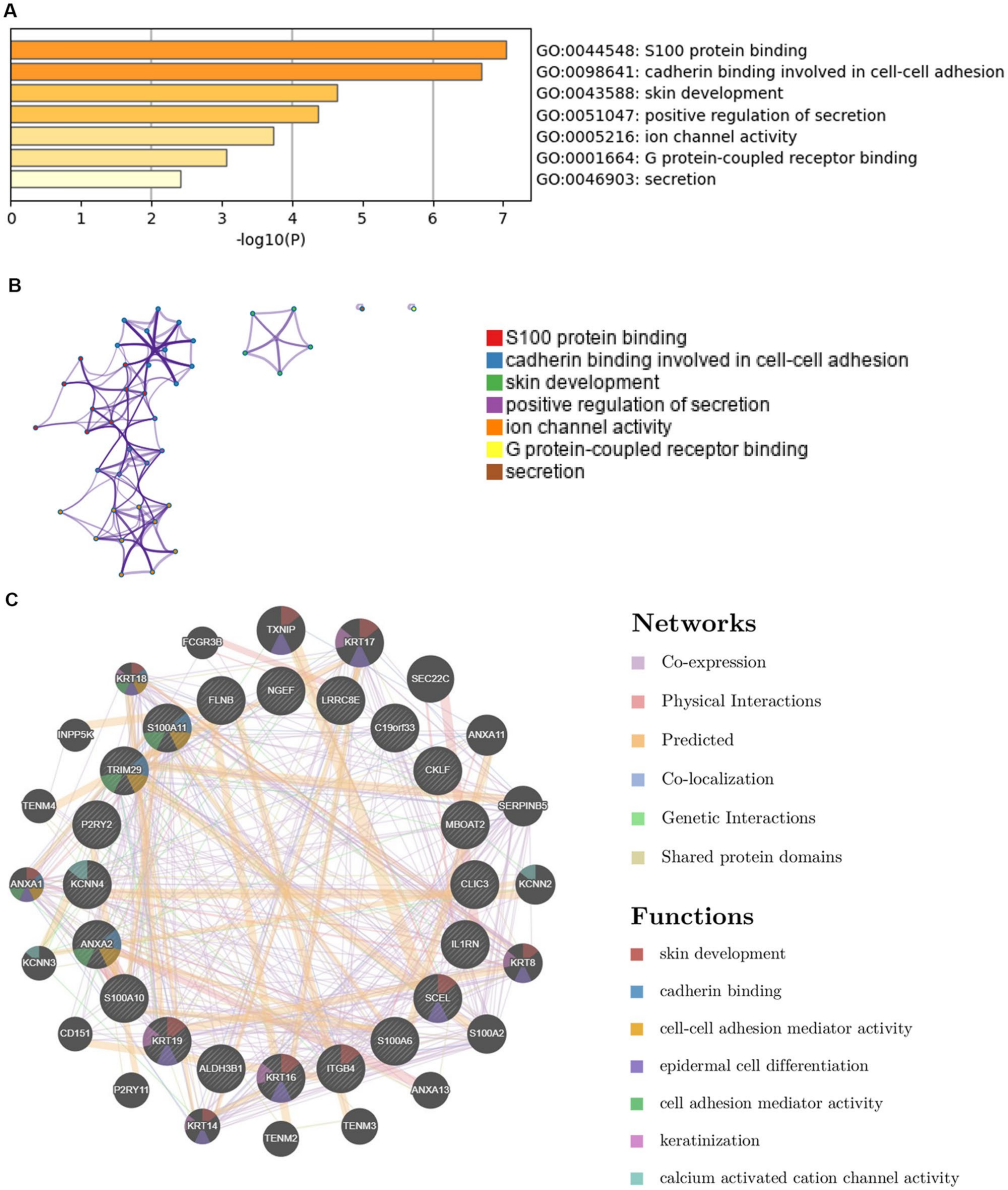
Functional annotations and interaction networks analyzed for *MMP28* and its similar genes. (**A**) GO enriched terms, colored by *P* values using Metascape. (**B**) Network of GO enriched terms colored by cluster-ID, where nodes sharing the same cluster-ID are typically close to each other using Metascape. (**C**) Twenty similar genes and their co-expression genes analyzed using GeneMANIA. The colors of lines represent different types of relationships. The colors inside the gene dots illustrate functions that these genes were involved in.

To understanding the function of MMP28, using the UALCAN database, we explored a list of co-expressed genes in PDAC that may play a synergistic role in the progression of pancreatic cancer. Enrichment analysis results based on Metascape revealed that those similar genes were preferentially involved in S100 protein binding, cadherin binding involved in cell–cell adhesion, and skin development (Fig. 5). Furthermore, the GeneMANIA database was used to predict the related networks and functions among these genes. The results demonstrated that co-expression (61.58%), physical interactions (20.61%), and predicted (13.72%) as major relationships among 20 similar genes. Other related networks, including co-localization, genetic interactions, and shared protein domains were 2.64%, 0.76%, and 0.70%, respectively. The functions of MMP28 and co-expressed genes were mainly correlated with skin development, cadherin binding, cell–cell adhesion mediator activity, epidermal cell differentiation, cell adhesion mediator activity, keratinization, calcium activated cation channel activity.

### GSEA analysis of *MMP28* in PDAC

**Figure 6:**
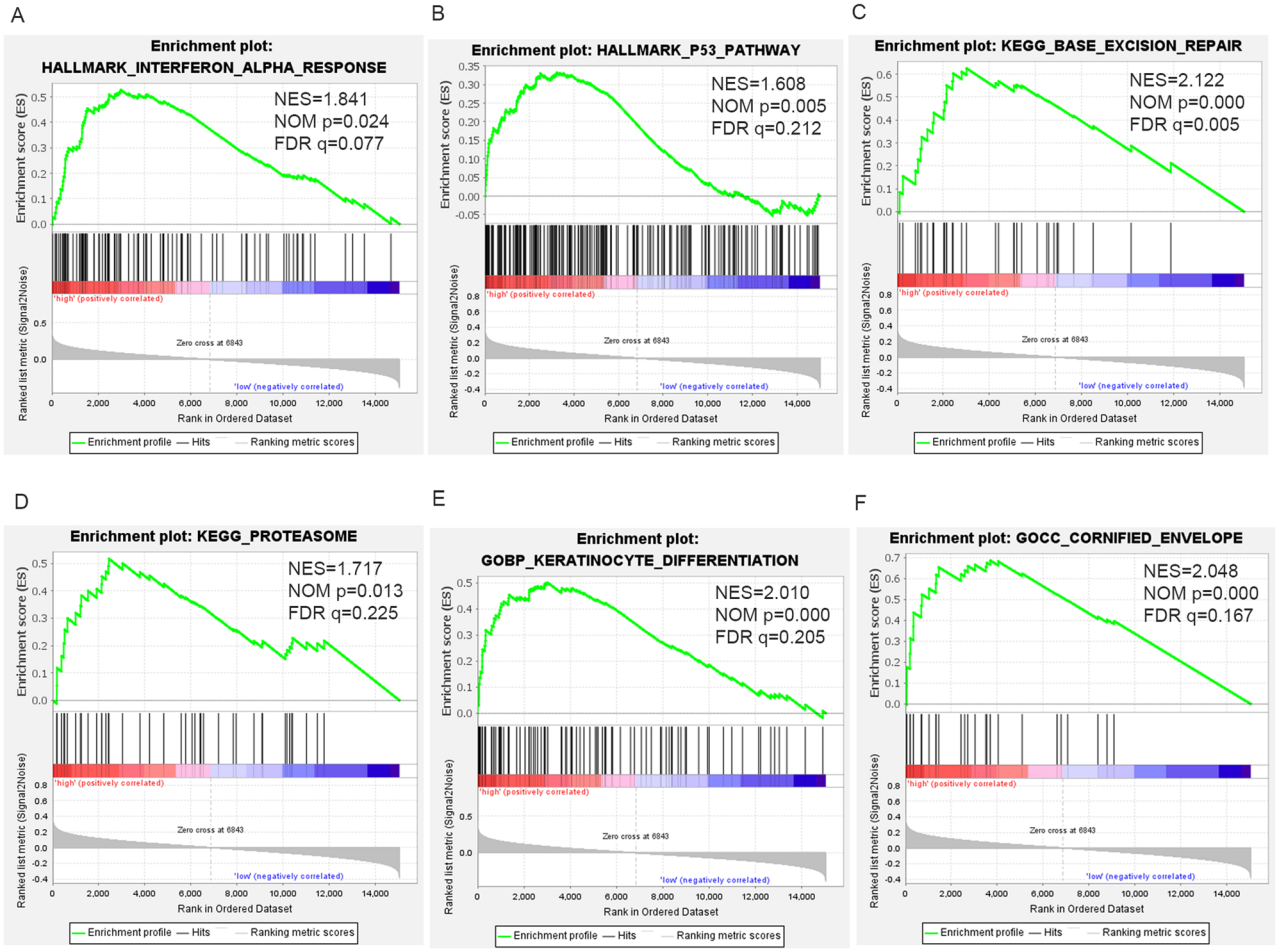
GSEA analysis of high *MMP28* expression in PDAC. Enrichment pathway of *MMP28* high expression group in (**A**,**B**) Hallmark, (**C**,**D**) C2 KEGG, and (**E**,**F**) C5 GO. *NES* Normalized enrichment score, *NOM* Nominal, *FDR* False discovery rate. Gene sets with NOM *P*-value < 0.05 and FDR q-value < 0.25 are considered significant.

To further explore the potential biological mechanism by which *MMP28* influenced the prognosis of PDAC, we conducted GSEA enrichment analysis. In Hallmark gene-set analysis, 27 gene sets were upregulated in the high MMP28 expression phenotype group. Two gene sets were significant at FDR q value < 0.25. Two gene sets were significantly enriched at NOM *P* value < 0.05, and 1 gene set are significantly enriched at NOM *P* value < 0.01. The most significant signaling pathways which met the criteria (NOM *P* value < 0.05 and FDR q value < 0.25) were interferon_alpha_response and P53_pathway (Fig. 6A,B). In KEGG analysis, 79 gene sets were upregulated in high expression group of *MMP28*. Two gene sets were found significant at FDR q value < 0.25. Five gene sets were significantly enriched at NOM *P* value < 0.05, and 1 gene set were significantly enriched at NOM *P* value < 0.01. As shown in Fig. 6C,D, the most significant enrichment pathways of KEGG analysis were base_excision_repair, proteasome. In GO analysis, 1684 gene sets were upregulated in the high MMP28 expression phenotype. Of them, 29 gene sets were considered significant at FDR q value < 0.25.149 gene sets were significantly enriched at NOM* P* value < 0.05, and 86 gene sets were significantly enriched at NOM *P* value < 0.01. GO analysis for up-regulated *MMP28* were mainly enriched in keratinocyte differentiation and cornified_envelope (Fig. 6E,F).

### Correlations between *MMP28* expression and immune infiltration levels in TIMER and TISCH

The tumor microenvironment (TME) plays a major role in tumor initiation, development, chemotherapy resistance, and cancer recurrence and metastasis. We assessed the correlation between *MMP28* expression and immune cell infiltration through the TIMER platform. Expression of *MMP28* was non-significant in all kinds of immune cell infiltration in PDAC (Fig. S4).

**Figure 7:**
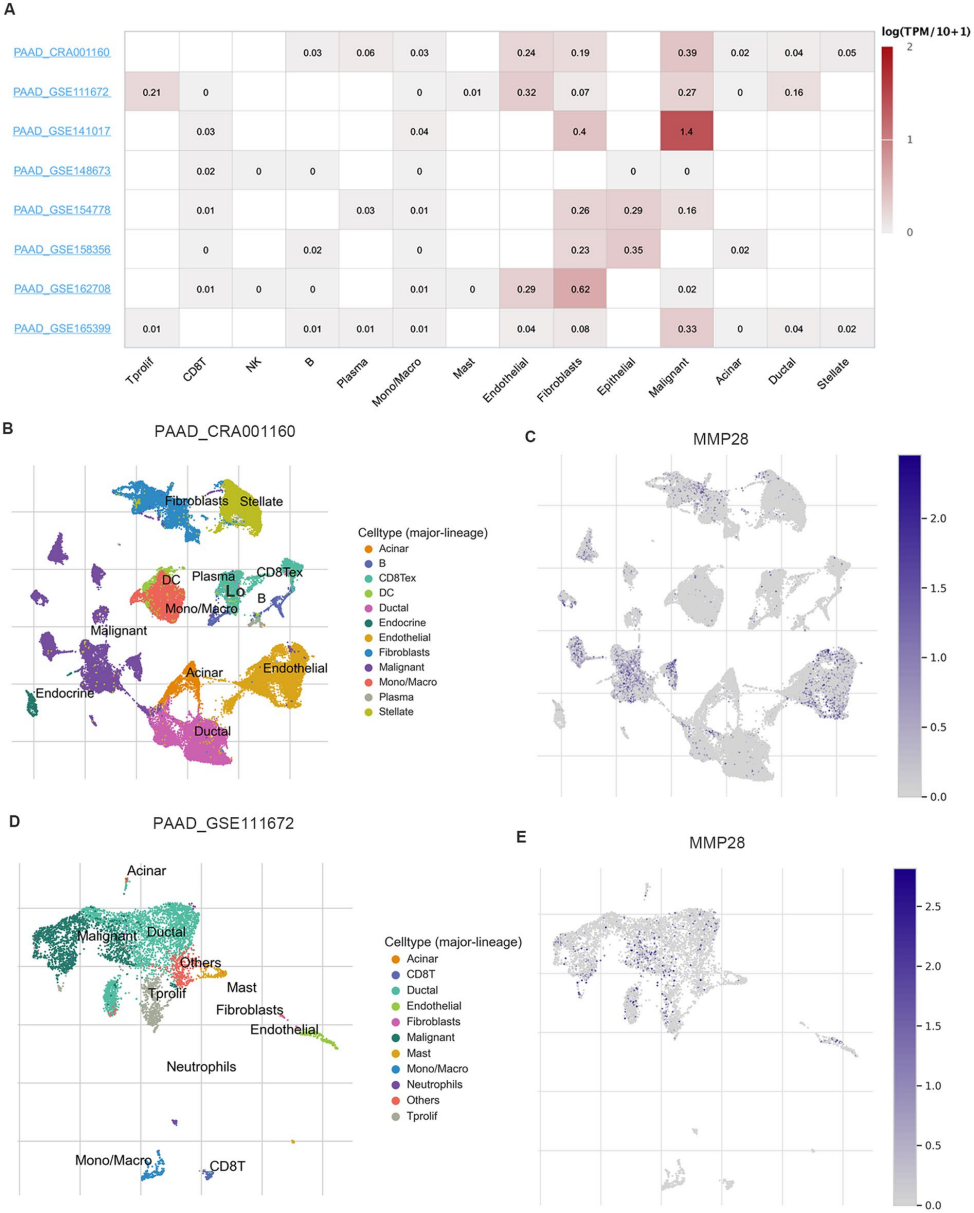
Correlations between MMP28 expression and immune infiltration levels in TISCH. (**A**) The heatmap displays average expression of *MMP28* (logTPM) in tumor microenvironment-related cells across PDAC datasets (TISCH). The color indicates the expression level of the gene. (**B**–**E**) Expression of *MMP28* at single-cell resolution in PAAD_ CRA001160 and PAAD_GSE111672.

To further validate the correlation between *MMP28* and immune infiltration in PDAC, we analyzed eight single-cell sequencing datasets of the TISCH database to evaluate *MMP28* expression in TME-related cells. The *MMP28* was mainly overexpressed in malignant tumor cells and stromal cells, while expression in immune cells was low (Fig. 7A). For instance, in the PAAD_ CRA001160 dataset, *MMP28* was mainly expressed in malignant cells, endothelial cells, and fibroblasts cells in the PDAC cell microenvironment (Fig. 7B,C). The *MMP28* expression level was relatively low for the immune cell components of TME, plasma cells, B cells, NK cells, CD8Tex cells, and Mono/Macro cells. The PAAD_GSE111672 was divided into nine cell types; the UMAP plots showed that *MMP28* expression level remained higher in malignant and endothelial cells compared to immune cells (Fig. 7D–,E). These results are consistent with those derived from the TIMER website.

### Expression of *MMP28* in pancreatic cancer cell lines

**Figure 8:**
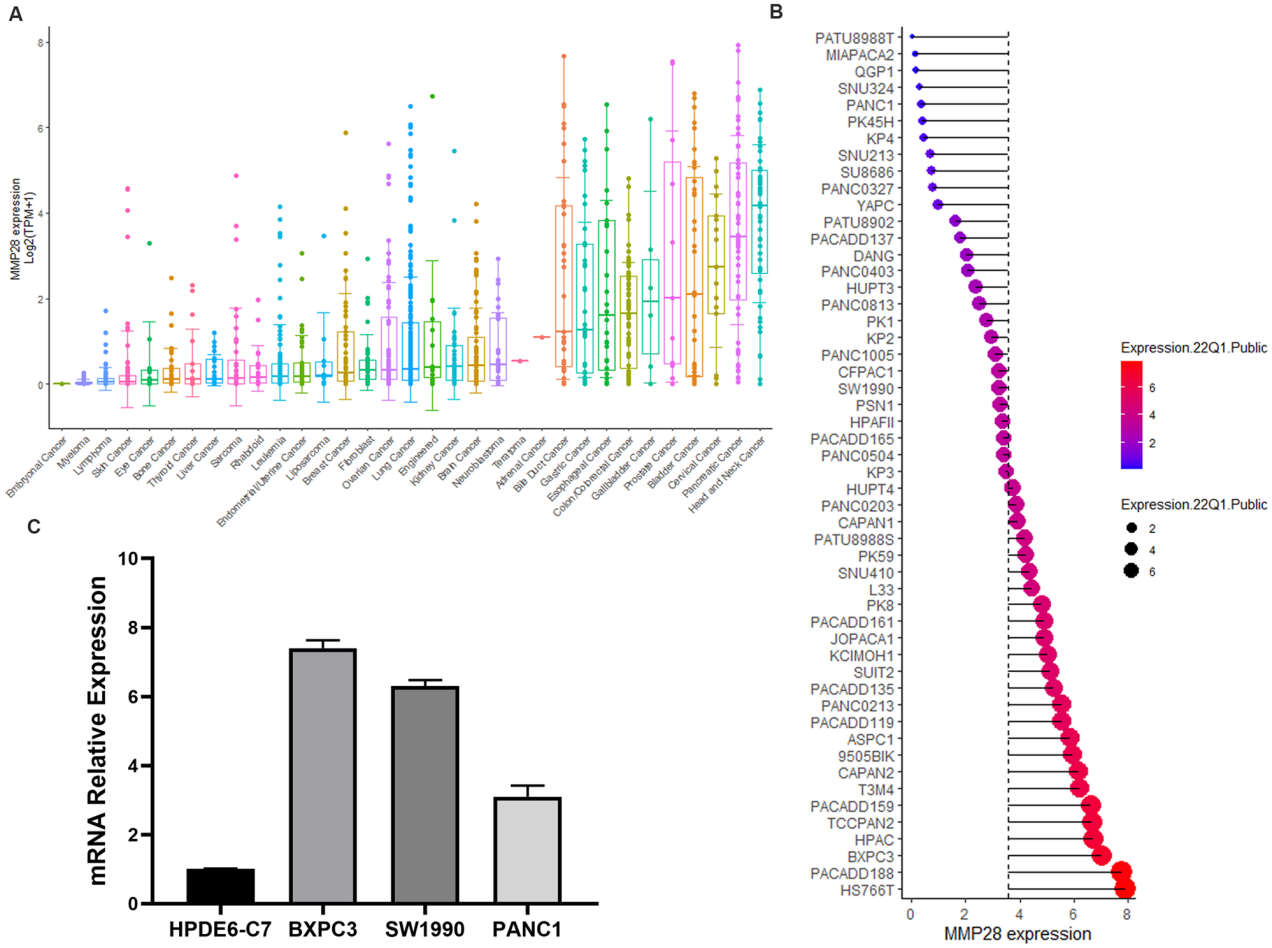
*MMP28* expression profile. (**A**,**B**) *MMP28* expression levels in various cancer cell lines and different pancreatic cancer cell lines using data from the CCLE database. (**C**) RT-qPCR was used to determine the expression pattern of *MMP28* in pancreatic cancer cell lines and normal pancreatic cells.

We then further explored the *MMP28* expression at the cellular level using the CCLE database. The gene expression profile of *MMP28* in various cancer cell lines and different pancreatic cancer cell lines is displayed in Fig. 8. Compared with other tumor cell lines, the mRNA expression level of *MMP28* was high in pancreatic cancer cell lines (Fig. 8A,B). Next, we measured expression of *MMP28* in pancreatic cancer cell lines and normal pancreatic cells using RT-qPCR. We found higher expressions of *MMP28* in BxPC-3 and SW1990 compared to that in hTERT-HPNE (Fig. 8C). In conclusion, the *MMP28* expression levels in most pancreatic cancer cell lines were higher than in the normal pancreatic cell line.

## Discussion

Previous studies reported that multiple members of the *MMP* family were associated with the progression of pancreatic cancer. For example, Li et al. reported that activated pancreatic stellate cells promoted the expression of *MMP2* in pancreatic carcinoma, and *MMP2* expression was positively correlated with lymph node metastasis and invasion of surrounding tissues and organs, but not with distant metastasis^32^. A study among 141 PDAC patients showed a positive tumoral MMP8 stain, and a low plasma CRP level predicted a favorable prognosis; MMP8 expression in the tumor could be considered as an independent positive prognostic factor for PDAC^33^. High levels of MMP7 in PDAC tissues were correlated with both tumor metastasis and one-year survival rate^34^. However, a comprehensive bioinformatic analysis for expression of all MMPs, their biological function, and underlying mechanism in pancreatic carcinogenesis has yet to be performed. In the current study, we examined the mRNA expression level of all 24 *MMP*s in PDAC and evaluated their prognostic value. Five highly expressed *MMP*s were significantly associated with worse survival outcomes in PDAC patients. Further, multivariate analysis showed that *MMP28* was an independent prognostic risk factor for PDAC. For in vitro experiments, *MMP28* was significantly up-regulated in PDAC cell lines. Taken together, the evidence strongly suggests that *MMP28* has potential as a prognostic biomarker for pancreatic cancer patients.

The gene *MMP28* (encoding epilysin) belongs to the *MMP19* subfamily. It was initially cloned from keratinocyte, testis, and mixed tumor cDNA libraries^35,36^. Among normal tissues, *MMP28* is relatively highly expressed in lung, testis, small intestine, and skin tissues^36–38^, and can participate in various pathophysiological processes such as inflammatory reaction, embryonic development, and angiogenesis^35,39,40^. Like other *MMP*s, aberrant upregulation of *MMP28* has been reported in several carcinomas, including colorectal cancer^41^, gastric carcinoma^42,43^, and hepatocellular carcinoma^44^. Our current study demonstrated that *MMP28* overexpression in tumor tissues was closely related to a poor outcome in PDAC patients. Further analysis of the correlation between *MMP28* expression and patient clinicopathological parameters showed that the higher the histological tumor grade, the higher was the expression of *MMP28*, implying its involvement in PDAC progression. However, there was no significant correlation between *MMP28* expression and tumor stage, possibly due to the uneven distribution of tumor patients in different stages in TCGA database. Next, clinical samples need to be collected to verify the correlation between *MMP28* expression and other clinical parameters.

Molecular pathology research and genome analysis have shown that the accumulation of various inherited or acquired gene mutations is crucial to the incidence and progression of early lesions induced by PDAC. There are four genes known to have a high mutation rate in PDAC: *KRAS*, *TP53*, *CDKN2A*, and *SMAD4*^45^. Among them, the tumor suppressor *p53* has a mutation rate of approximately 60–70% and regulates tumor initiation and invasion in pancreatic cancer cells^45,46^. Our study showed that *MMP28* was significantly up-regulated in patients with *TP53* mutations. The GSEA results showed that *MMP28* expression was mainly raised in the P53 signaling pathway. In a recent study, based on orthotopic mouse PDAC xenografts and human tumor samples, the presence of missense *TP53* mutations selectively reduced the infiltration of cytotoxic CD8 + T cells into PDAC tumors and promoted tumor microenvironment fibrosis^47^. Previous study reported that the oncogenic protein *PARP1* was involved in transcriptional up-regulation of *MMP28* via the STAT3-MMP7 axis in PDAC^48^. Khalid, M et al. reported that *MMP28*, as a downstream gene influenced by *RACGAP1*, was involved in the pathogenesis of PDAC^49^. More experimental studies are needed to explore the interaction between MMP28 and tumor-related signaling pathways during pancreatic carcinogenesis.

The characteristic highly inflammatory and desmoplastic TME are the main obstacles to effective PDAC treatment. Recently, a single-cell RNA sequencing (scRNA-seq) approach was applied to explore dynamic changes of tumor microenvironment during PDAC progression^50^. In this study, we established the expression profile of *MMP28* in various cell subtypes using a scRNA-seq platform. The results indicated higher expression levels of *MMP28* in stromal and malignant tumor cells compared with immune cells. These findings are consistent with the results from the TIMER website. The above results indicate that *MMP28* might be a novel gene associated with malignant cells and stromal cell infiltration, thus influencing TME in PDAC.

However, there were some deficiencies in our research. First, the study lacked an external clinical cohort validation set and experimental validation. Second, the uneven sample distribution among different groups affected the follow-up analysis between MMP28 and clinical parameters. Third, several computational models have been applied to explore genetic markers and related diseases during recent years, such as SWATH-MS-based network modeling, DMFGAM prediction model, deep learning algorithm based on GCNAT and mRNA-driven protein liquid–liquid phase separation model^51–54^, and we can refer to these models to verify our research results. The interaction prediction research in various fields of computational biology will provide valuable insights for finding potential therapeutic targets. Nevertheless, we identified five highly expressed MMPs were significantly associated with worse survival outcomes in PDAC patients using bioinformatics analysis. Among the five hub genes, we found that overexpression of *MMP28* was significantly correlated with poor PDAC patient prognosis. Bioinformatics, as an important tool for tumor research, provides prediction and guidance for subsequent experimental research. In future studies, we will conduct in vitro and in vivo experiments to verify the cell molecules and signal pathways interacting with *MM28*, to provide new insights into the mechanisms of PDAC development and therapeutic strategies.

## Conclusions

Our findings suggest that five highly expressed *MMPs (MMP1*, *MMP3*, *MMP11*, *MMP14*, and *MMP28*)) were significantly associated with worse survival outcomes in PDAC patients. Among them, *MMP28* was an independent prognostic risk factor to predict the prognosis of patients with PDAC. The *MMP28* may also play a vital role in the PDAC tumor microenvironment by regulating malignant and stromal cells, suggesting that *MMP28* may provide novel therapeutic target for PDAC treatments.

### Supplementary Information

Supplementary Information.

## Data Availability

The datasets analyzed during the current study are available from the Gene-Expression Omnibus (GEO; https://www.ncbi.nlm.nih.gov/geo/) and TCGA data portal (https://tcga-data.nci.nih.gov/tcga/) repository.
