# Supplementary material for: Identification of novel therapeutic target and prognostic biomarker in matrix metalloproteinase gene family in pancreatic cancer
Source: Sci Rep. 2023 Oct 11;13:17211. doi: 10.1038/s41598-023-44506-8 (PMC10567842; doi:10.1038/s41598-023-44506-8)
Supplement: Supplementary file 1 — Supplementary Information. [file 41598_2023_44506_MOESM1_ESM.docx]

Figure S1: The overall flowchart for the strategies and methods used in this study. [19,20], [21], [22,23], [24],[25], [26,27] and [28] represent corresponding references in the manuscript.


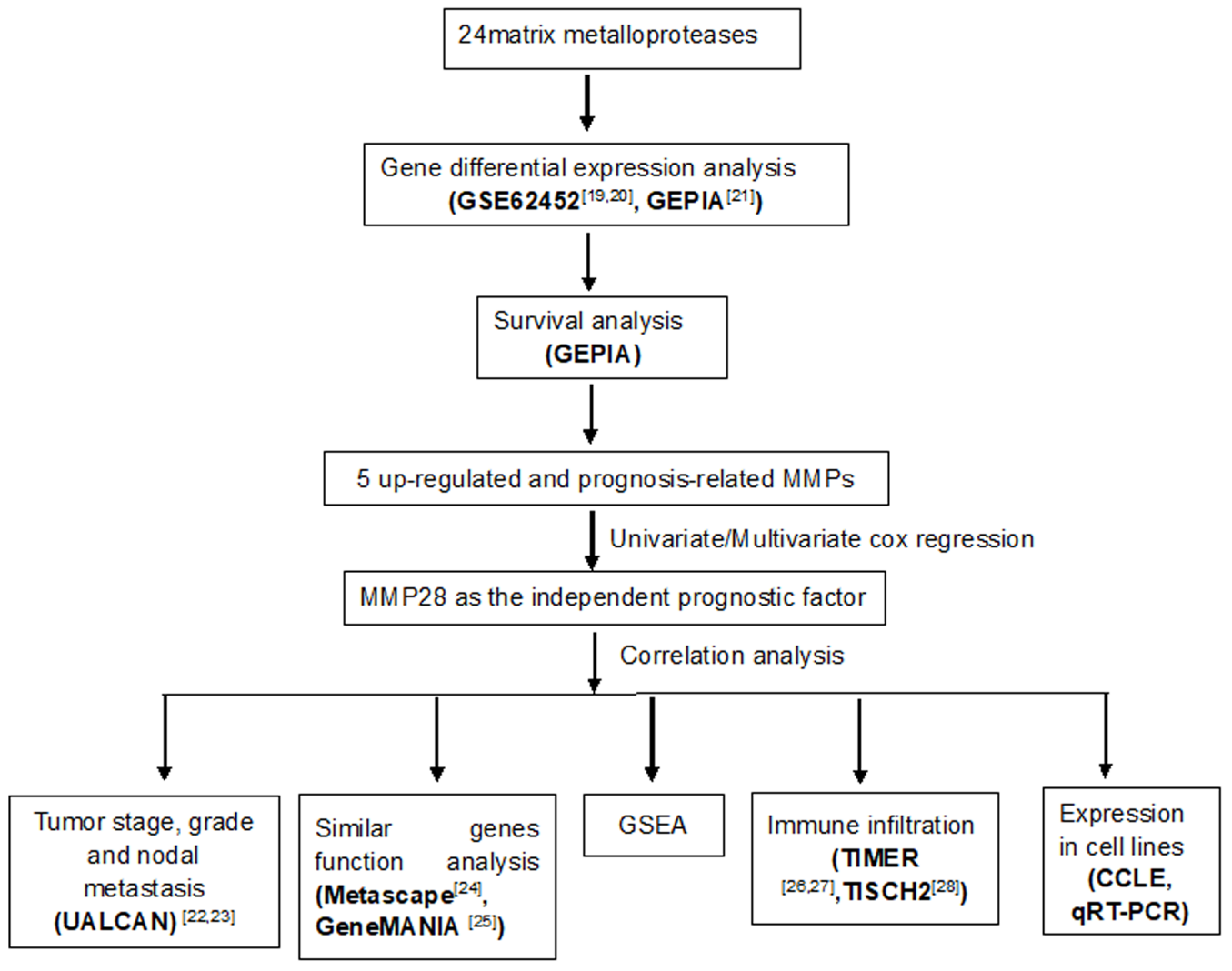


Figure S2: The differential expression of *MMP*s family in pancreatic cancer tissue and adjacent pancreatic non-tumor tissue in GSE62452.


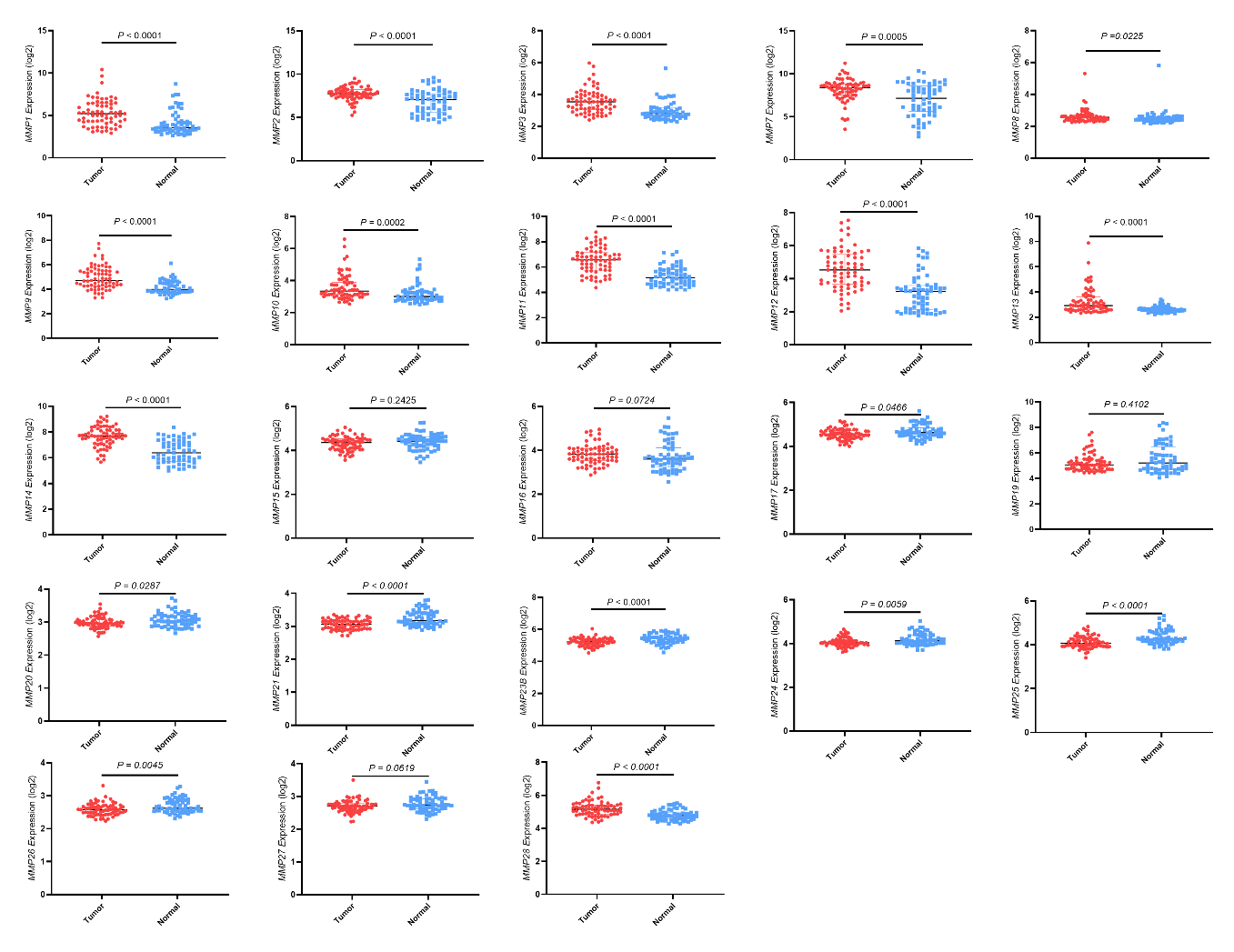


Figure S3: Overall survival (OS) and disease-free survival (DFS) of five *MMP*s (*MMP1, MMP3, MMP11, MMP14, MMP28*) in patients with PDAC.
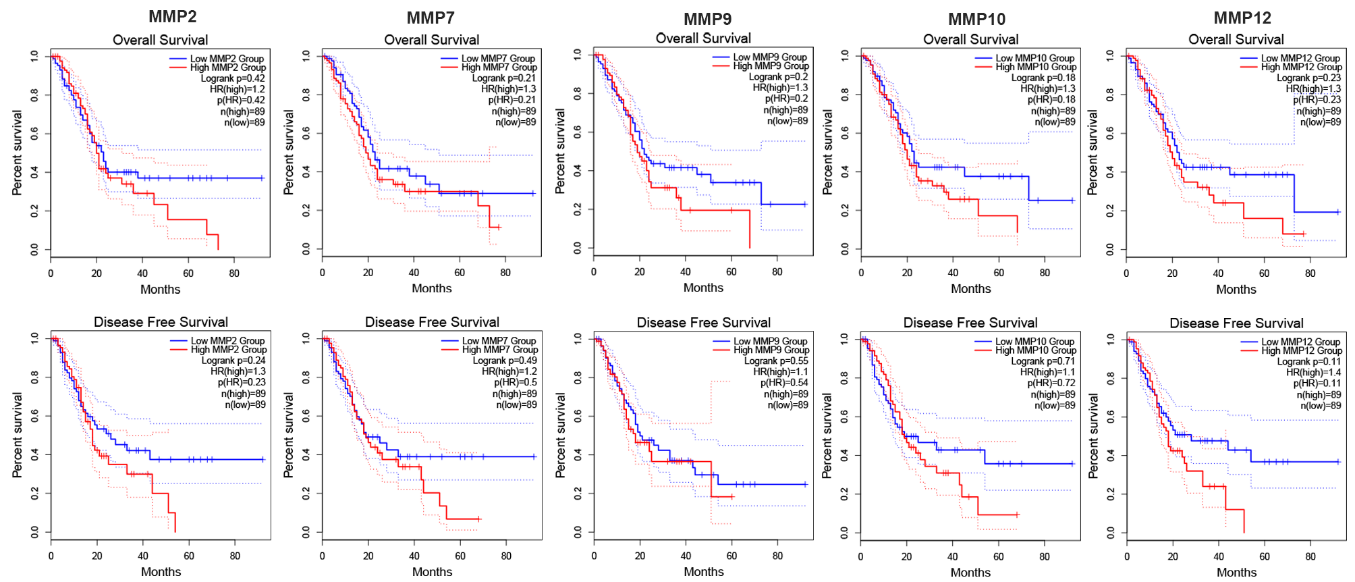


Figure S4: The correlation of 6 immunocytes with *MMP28* expression was analyzed by TIMER.


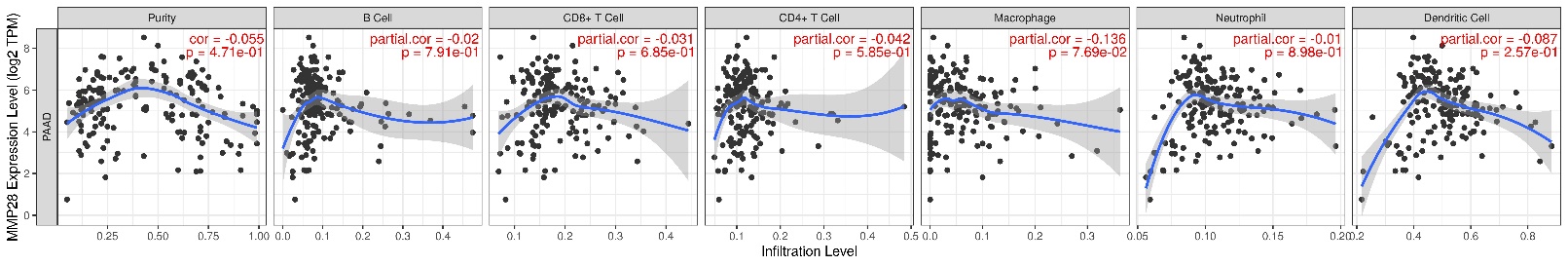


Table S1. Primers used for PCR amplification.

| Gene | Primer sequence |
| --- | --- |
| MMP28 | Forward: 5ʹ- TCCCACCTCCACTCGATTCAG-3ʹ |
|  | Reverse: 5ʹ-GCCGCATAACTGTTGGTATCT-3ʹ |
| GAPDH | Forward: 5ʹ-ACAACTTTGGTATCGTGGAAGG-3ʹ |
|  | Reverse:5ʹ- GCCATCACGCCACAGTTTC-3ʹ |

| Table S2. The corrections of 5 prognosis-related *MMP*s and tumor stage, lymph node metastasis and tumor grade. | | | | | | |
| --- | --- | --- | --- | --- | --- | --- |
|  | Tumor stage | | Tumor Grade | | Nodal metastases | |
|  | Comparison | Statistical significance | Comparison | Statistical significance | Comparison | Statistical significance |
| MMP1 |  |  |  |  |  |  |
|  | Normal-vs-Stage1 | 0.3142 | Normal-vs-Grade 1 | 0.8942 | Normal-vs-N0 | 0.0070 |
|  | Normal-vs-Stage2 | 0.0001 | Normal-vs-Grade 2 | 0.7464 | Normal-vs-N1 | 0.0003 |
|  | Normal-vs-Stage3 | 0.1345 | Normal-vs-Grade 3 | 0.9289 | N0-vs-N1 | 0.3827 |
|  | Normal-vs-Stage4 | 0.0763 | Normal-vs-Grade 4 | 1.0000 |  |  |
|  | Stage1-vs-Stage2 | 0.0005 | Grade 1-vs-Grade 2 | 0.4760 |  |  |
|  | Stage1-vs-Stage3 | 0.3908 | Grade 1-vs-Grade 3 | 0.6811 |  |  |
|  | Stage1-vs-Stage4 | 0.1207 | Grade 1-vs-Grade 4 | 0.8942 |  |  |
|  | Stage2-vs-Stage3 | 0.0039 | Grade 2-vs-Grade 3 | 0.0550 |  |  |
|  | Stage2-vs-Stage4 | 0.3466 | Grade 2-vs-Grade 4 | 0.7464 |  |  |
|  | Stage3-vs-Stage4 | 0.1493 | Grade 3-vs-Grade 4 | 0.9289 |  |  |
| MMP3 |  |  |  |  |  |  |
|  | Normal-vs-Stage1 | 0.2996 | Normal-vs-Grade 1 | 0.2772 | Normal-vs-N0 | 0.3781 |
|  | Normal-vs-Stage2 | 0.0950 | Normal-vs-Grade 2 | 0.9617 | Normal-vs-N1 | 0.1124 |
|  | Normal-vs-Stage3 | 0.3475 | Normal-vs-Grade 3 | 0.5912 | N0-vs-N1 | 0.2470 |
|  | Normal-vs-Stage4 | 0.2480 | Normal-vs-Grade 4 | 1.0000 |  |  |
|  | Stage1-vs-Stage2 | 0.4157 | Grade 1-vs-Grade 2 | 0.2774 |  |  |
|  | Stage1-vs-Stage3 | 0.5572 | Grade 1-vs-Grade 3 | 0.5486 |  |  |
|  | Stage1-vs-Stage4 | 0.7995 | Grade 1-vs-Grade 4 | 0.2772 |  |  |
|  | Stage2-vs-Stage3 | 0.9365 | Grade 2-vs-Grade 3 | 0.2278 |  |  |
|  | Stage2-vs-Stage4 | 0.2645 | Grade 2-vs-Grade 4 | 0.9617 |  |  |
|  | Stage3-vs-Stage4 | 0.5200 | Grade 3-vs-Grade 4 | 0.5912 |  |  |
| MMP11 |  |  |  |  |  |  |
|  | Normal-vs-Stage1 | 0.4040 | Normal-vs-Grade 1 | 0.8754 | Normal-vs-N0 | 0.9078 |
|  | Normal-vs-Stage2 | 0.8247 | Normal-vs-Grade 2 | 0.3811 | Normal-vs-N1 | 0.8197 |
|  | Normal-vs-Stage3 | 0.6125 | Normal-vs-Grade 3 | 0.4043 | N0-vs-N1 | 0.5723 |
|  | Normal-vs-Stage4 | 0.3716 | Normal-vs-Grade 4 | 1.0000 |  |  |
|  | Stage1-vs-Stage2 | 0.2636 | Grade 1-vs-Grade 2 | 0.0000 |  |  |
|  | Stage1-vs-Stage3 | 0.4492 | Grade 1-vs-Grade 3 | 0.0004 |  |  |
|  | Stage1-vs-Stage4 | 0.0469 | Grade 1-vs-Grade 4 | 0.8754 |  |  |
|  | Stage2-vs-Stage3 | 0.6144 | Grade 2-vs-Grade 3 | 0.9799 |  |  |
|  | Stage2-vs-Stage4 | 0.5055 | Grade 2-vs-Grade 4 | 0.3811 |  |  |
|  | Stage3-vs-Stage4 | 0.7663 | Grade 3-vs-Grade 4 | 0.4043 |  |  |
| MMP14 |  |  |  |  |  |  |
|  | Normal-vs-Stage1 | 0.3925 | Normal-vs-Grade 1 | 0.6266 | Normal-vs-N0 | 0.73096 |
|  | Normal-vs-Stage2 | 0.9282 | Normal-vs-Grade 2 | 0.7874 | Normal-vs-N1 | 0.92396 |
|  | Normal-vs-Stage3 | 0.8335 | Normal-vs-Grade 3 | 0.8149 | N0-vs-N1 | 0.32888 |
|  | Normal-vs-Stage4 | 0.9992 | Normal-vs-Grade 4 | 1.0000 |  |  |
|  | Stage1-vs-Stage2 | 0.0044 | Grade 1-vs-Grade 2 | 0.0000 |  |  |
|  | Stage1-vs-Stage3 | 0.3262 | Grade 1-vs-Grade 3 | 0.0001 |  |  |
|  | Stage1-vs-Stage4 | 0.0529 | Grade 1-vs-Grade 4 | 0.6266 |  |  |
|  | Stage2-vs-Stage3 | 0.7441 | Grade 2-vs-Grade 3 | 0.5597 |  |  |
|  | Stage2-vs-Stage4 | 0.9154 | Grade 2-vs-Grade 4 | 0.7874 |  |  |
|  | Stage3-vs-Stage4 | 0.7902 | Grade 3-vs-Grade 4 | 0.8149 |  |  |
| MMP28 |  |  |  |  |  |  |
|  | Normal-vs-Stage1 | 0.3925 | Normal-vs-Grade 1 | 0.6266 | Normal-vs-N0 | 0.73096 |
|  | Normal-vs-Stage2 | 0.9282 | Normal-vs-Grade 2 | 0.7874 | Normal-vs-N1 | 0.92396 |
|  | Normal-vs-Stage3 | 0.8335 | Normal-vs-Grade 3 | 0.8149 | N0-vs-N1 | 0.32888 |
|  | Normal-vs-Stage4 | 0.9992 | Normal-vs-Grade 4 | 1.0000 |  |  |
|  | Stage1-vs-Stage2 | 0.0044 | Grade 1-vs-Grade 2 | 0.0000 |  |  |
|  | Stage1-vs-Stage3 | 0.3262 | Grade 1-vs-Grade 3 | 0.0001 |  |  |
|  | Stage1-vs-Stage4 | 0.0529 | Grade 1-vs-Grade 4 | 0.6266 |  |  |
|  | Stage2-vs-Stage3 | 0.7441 | Grade 2-vs-Grade 3 | 0.5597 |  |  |
|  | Stage2-vs-Stage4 | 0.9154 | Grade 2-vs-Grade 4 | 0.7874 |  |  |
|  | Stage3-vs-Stage4 | 0.7902 | Grade 3-vs-Grade 4 | 0.8149 |  |  |
